# LEAM vs. BEAM vs. CLV Conditioning Regimen for Autologous Stem Cell Transplantation in Malignant Lymphomas. Retrospective Comparison of Toxicity and Efficacy on 222 Patients in the First 100 Days After Transplant, On Behalf of the Romanian Society for Bone Marrow Transplantation

**DOI:** 10.3389/fonc.2019.00892

**Published:** 2019-09-10

**Authors:** Andrei Colita, Anca Colita, Horia Bumbea, Adina Croitoru, Carmen Orban, Lavinia Eugenia Lipan, Oana-Gabriela Craciun, Dan Soare, Cecilia Ghimici, Raluca Manolache, Ionel Gelatu, Ana-Maria Vladareanu, Sergiu Pasca, Patric Teodorescu, Delia Dima, Anca Lupu, Daniel Coriu, Ciprian Tomuleasa, Alina Tanase

**Affiliations:** ^1^Department of Hematology, Carol Davila University of Medicine and Pharmacy, Bucharest, Romania; ^2^Department of Stem Cell Transplantation, Coltea Clinical Hospital, Bucharest, Romania; ^3^Department of Pediatrics, Carol Davila University of Medicine and Pharmacy, Bucharest, Romania; ^4^Department of Stem Cell Transplantation, Fundeni Clinical Institute, Bucharest, Romania; ^5^Department of Stem Cell Transplantation, University Hospital, Bucharest, Romania; ^6^Department of Hematology, Research Center for Functional Genomics and Translational Medicine, Iuliu Hatieganu University of Medicine and Pharmacy, Cluj Napoca, Romania; ^7^Department of Hematology, Ion Chiricuta Clinical Cancer Center, Cluj Napoca, Romania; ^8^Department of Hematology, Iuliu Hatieganu University of Medicine and Pharmacy, Cluj Napoca, Romania; ^9^Department of Hematology, Fundeni Clinical Institute, Bucharest, Romania

**Keywords:** relapsed/refractory lymphoma, autologous stem cell transplantation, conditioning chemotherapy, retrospective analysis, real-life data

## Abstract

High-dose chemotherapy (HDT) followed by autologous hematopoietic stem cell transplantation (ASCT) is widely used in patients with malignant lymphomas. In Europe over 8,000 ASCTs for lymphoma were performed out of a total of 40,000 transplants according to the European Bone Marrow Transplant (EBMT) activity survey in 2017. ASCT is considered the standard treatment for eligible patients failing to achieve remission after first line chemotherapy or patients with relapsed or refractory lymphomas, including classical Hodkin's lymphoma, diffuse large B-cell lymphoma, mantle cell lymphoma, and follicular lymphoma, as well as consolidation therapy in first remission in mantle cell lymphoma. BEAM (BCNU/carmustine, etoposide, cytarabine, and melphalan) is the most commonly used conditioning regimen for ASCT in patients with relapsed/refractory (R/R) lymphomas in Europe, whereas the CBV (cyclophosphamide, BCNU, and etoposide) regimen is also widely used in North America. Recently, concerns regarding BCNU toxicity as well as restricted availability of BCNU and melphalan has determined an increasing number of transplant centers to use alternative conditioning regimens. Currently, only a few comparative studies, most of them retrospective, between different conditioning protocols regarding efficacy and toxicity have been published. Thus, in the current manuscript, we report the experience of 2 transplant centers in ASCT in R/R lymphomas with three types of conditioning: BEAM, CLV (cyclophosphamide, lomustine, etoposide) and LEAM (lomustine, etoposide, cytarabine, and melphalan), with the aim to evaluate the results of alternative conditioning regimens using lomustine (LEAM and CLV) and compare them with the standard BEAM regarding early toxicity, engraftment, and transplant related mortality (TRM). All patients developed grade IV neutropenia, anemia with/without transfusion necessity. Severe thrombocytopenia with transfusion requirements is reported in most cases. Median time to platelet engraftment and neutrophil engraftment was 13 days (range) and 10 days (range), respectively. Gastrointestinal toxicity was the most common non-hematologic toxicity after all three conditioning regimens. Oral mucositis in various grades from I to IV was diagnosed in most cases. Other side effects include vomiting, diarrhea, colitis, and skin rash but with low severity grades. For the LEAM arm, one patient died after transplant, before engrafting, one patient didn't achieve platelet engraftment in day 100, one patient developed grade 3 upper gastrointestinal bleeding, one patient died (grade 5 toxicity) with acute renal failure, one patient developed hypoxic events up to grade 4 acute respiratory failure and one patient developed grade 3 itchy skin rash. For the CLV arm, one patient died after transplant, before engrafting, one patient developed grade 3 colitis, one patient with grade 3 hepatic cytolysis, one patient with cardiac toxicity followed by death (grade 5) caused by an acute myocardial infarction with ST elevation and one patient with pulmonary toxicity clinically manifested with grade 3 pleurisy. For the BEAM arm, one patient developed grade 3 cardiac toxicity with sinus bradycardia and afterwards grade 4 with acute pulmonary edema, three patients presented a grade 3 pruritic skin rash and two patients developed grade 3 seizures. In the present study we presented the differences that were observed between BEAM, LEAM, and CLV conditioning regimens offering clinical arguments for an SCT practitioner choice in the ideal situation, but also of choice for alternative regimens in the case that one regimen cannot be used.

## Introduction

In 2019, an estimated of approximately 82,000 lymphomas will be diagnosed in the Western world, with almost 21,000 deaths (1). Chemoimmunotherapy is the first line therapy of choice for most of these cases, especially for aggressive lymphomas, out of which up to 30% of all B-cell non-Hodgkin's lymphomas (NHL) don't achieve a complete remission (CR) with standard induction treatment, as is the case for R-CHOP (rituximab, cyclophosphamide, doxorubicin, vincristine, and prednisone) (2). The role of ASCT in the standard treatment of T cell lymphomas is less well defined than for relapsed B-cell lymphoma. A relapsed NHL is treated using salvage chemotherapy (ST) and HDT, followed by consolidation with an ASCT (3, 4).

For R/R lymphomas that undergo an ASCT, BEAM is a conditioning regimen option, being compared with LEAM (lomustine, etoposide, cytarabine, and melphalan) (5). The choice of conditioning chemotherapy has not been clarified by retrospective studies and there is no prospective data to support the use of one conditioning regimen over another. The replacement of BCNU with lomustine has been studied by Kothari et al. with no significant difference in transplant outcomes, although the number of patients included in this study was small. When comparing LEAM to CBV (cyclophosphamide, BCNU and etoposide) on 71 ASCT R/R lymphoma patients, dos Santos et al. have shown that mortality within the first 100 days was statistically favorable for LEAM when compared with CBV, suggesting less toxicity, but there was no statistically significant difference in PFS between the LEAM and CBV protocols. When the OS was evaluated, it was higher in the LEAM group (6). The number of patients was still limited. The CBV (cyclophosphamide, BCNU, etoposide) regimen is also widely used in North America (7). Over the last years, concerns regarding BCNU toxicity as well as restricted availability of BCNU and melphalan have determined an increasing number of transplant centers to use alternative conditioning regimens. Currently, there are only a few comparative studies, most of them retrospective, between different conditioning protocols regarding efficacy and toxicity (8).

As a limited number of studies have looked over the toxicity and efficacy of conditioning regimens on R/R lymphoma patients undergoing an ASCT, in the current manuscript we present a retrospective analysis on a large cohort (*n* = 222), comparing BEAM vs. LEAM vs. CLV (CBV with lomustine replacing BCNU).

## Patients and Methods

### Patients

The study was a retrospective analysis of the Romanian Society for Bone Marrow Transplantation. We identified 222 patients reported in the registry of the Fundeni Clinical Institute and the Coltea Clinical Hospital in Bucharest, Romania, as having undergone an ASCT for a R/R malignant lymphoma [both Hodgkin lymphomas (HL) as well as NHL] (Table 1). The inclusion criteria were date of ASCT between January 2012 and August 2017, diagnosis of HL, T-cell NHL, B-cell NHL and composite NHL, no previous stem cell procedures and conditioning with either LEAM, BEAM and CLV chemotherapy. We have included 34 patients from the pediatric ward and 188 patients from the adult ward. Functional imaging with 18-F-fluorodeoxyglucose (FDG)-positron emission tomography combined with computed tomography (PET-CT) was carried out in all cases, both before autologous SCT in order to optimize the outcome of the therapeutics with only patients in CR being eligible for SCT. PET-CT was also performed post-transplant, to evaluate the outcome of the therapy, according to previously published data (9–11).

**Table 1:** Baseline characteristics of the patients enrolled in the study.

**Characteristics**		***n* = 222**
Sex	Female	45.0% (*n* = 100)
	Male	55.0% (*n* = 122)
Median age (quartile 1, quartile 3) (years)		36.9 (25.9, 45.2)
Diagnostic	HL	56.3% (*n* = 125)
	NHL-B	32.9% (*n* = 73)
	NHL-T	9.0% (*n* = 20)
	Composite NHL	1.8% (*n* = 4)
Stage at diagnosis	I/II	25.7% (*n* = 57)
	III/IV	74.3% (*n* = 165)
Number of previous chemotherapy lines	1 or 2	52.3% (*n* = 116)
	3	30.6% (*n* = 68)
	4 or more	17.1% (*n* = 38)
Status before autoHSCT	CR	47.3% (*n* = 105)
	PR	38.3% (85%)
	SD	0.9% (*n* = 2)
	PD	13.1% (*n* = 29)

Dosages for BEAM conditioning regimen were according to Caballero et al. (12), in LEAM, Lomustine 200 mg/m^2^ at day −7 replaced BCNU, whereas in CVL dosages were according to Majolno et al. with Lomustine 300 mg/m^2^ at day −6 replacing BCNU (Table 2) (13–15).

**Table 2:** Detailed dosages for the conditioning chemotherapy.

**Conditioning chemotherapy regimen**	**Doses**	**References**
BEAM	BCNU (carmustine) 300 mg/m2 (total dose) i.v. on day-6, etoposide 800 mg/m2 (total dose) i.v. divided over 4 days from days−5 to−2, ara-C (cytarabine) 1,600 mg/m2 (total dose) i.v. twice daily divided over 4 days from days−5 to−2, and melphalan 140 mg/m2 (total dose) i.v. on day-1	(13)
LEAM	CCNU (Lomustine) 200 mg/m2 p.o. on day-6, etoposide 800 mg/m2 (total dose) i.v. divided over 4 days from days−5 to−2, ara-C (cytarabine) 1,600 mg/m2 (total dose) i.v. twice daily divided over 4 days from days−5 to−2, and melphalan 140 mg/m2 (total dose) i.v. on day-1	(14)
CLV	Lomustine 300 mg/m^2^ p.o. on day-6, etoposide 800 mg/m2 (total dose) i.v. divided over 4 days from days−5 to−2, ara-C (cytarabine) 1,600 mg/m2 (total dose) i.v. twice daily divided over 4 days from days−5 to−2, and melphalan 140 mg/m2 (total dose) i.v. on day-1	(14)

The objective was to assess the hematological and extra-hematological toxicities of the three conditioning regimens, the NRM and OS of the three patient cohorts. Standard conditioning therapies for ASCT in lymphoma are currently thus BCNU-based and BEAM is frequently used in Europe, while CBV is frequently used in North America. Shortage of BCNU is a world-wide problem caused by limited availability of the alcoholic solvent needed for its preparation, leading to the development of other conditioning regimens for ASCT in patients with lymphoma. Lomustine (CCNU) is a potential substitute of BCNU and determines the use of alternative regimens, as is the case for LEAM and CLV. Melphalan-related problems on market required the emergence of new conditioning regimens without melphalan (e.g., CLV, LACE (lomustine, cytarabine (Ara-C), cyclophosphamide, etoposide. The current manuscript presents retrospective data in the real-life setting. In Romania, BCNU was not available and thus it was replaced with CCNU, turning BEAM conditioning chemotherapy into LEAM conditioning. The same situation was reported when melphalan was absent and thus an alternative for conditioning was CLV.

Neutrophil engraftment was defined as the first of the three consecutive days post-transplant where absolute neutrophil count reached > 0.5 × 109/l and platelet count >20 × 109/l, unsupported by transfusion and G-CSF more than 5 days from the first increase in the investigated parameters.

### Study Definitions

Disease status was defined as follows: CR was defined as the disappearance of tumor masses and disease-related symptoms; partial response (PR) was considered when measurable lesions decreased by at least 50%; disease status at transplant was considered to be “chemosensitive” if at least PR was achieved following the last course of chemotherapy, otherwise it was considered as “chemo resistant.” CR/PR1 refers to transplants performed in first response; CR/PR > 1 refers to transplants performed beyond first response. NRM included all deaths without a previously registered episode of relapse or progression. Relapse was defined as the occurrence of new sites of disease after a CR lasting for 3 months or longer, whereas progression was considered when CR had not been achieved. OS was defined as the time from transplant to death from any cause. Good performance status (PS) was defined as Karnofsky score >80% or ECOG score 0–1.

### Statistics

Data analysis was performed using R 3.5.1. Categorical data was represented as percent (absolute value). Shapiro-Wilk test was used to determine the normality of the distribution. Mean and standard deviation were used for normally distributed samples. Median, quartile 1 and quartile 3 were used for non-normally distributed samples. ANOVA was used for comparing more than 2 normally distributed groups. Kruskal-Wallis was used for comparing more than 2 non-normally distributed groups. If Kruskal-Wallis test was significant, we used pairwise Wilcox test with Benjamini-Hochberg correction. Welch *t*-test was used for comparing 2 normally distributed groups. Mann-Whitney-Wilcoxon test was used for comparing 2 non-normally distributed groups. Fisher test was used to compare categories. Kaplan-Meier curves were used to represent survival differences between the three conditioning regimens. For the multivariate survival analysis, we used a Cox proportional hazards model in which we included the variables that presented statistical significance in the univariate Cox proportional hazards model. A *p*-value under 0.05 was considered significant.

For the univariate analysis for RFS at 100 days we included age over 50, male sex, Hodgkin lymphoma diagnosis, Stage 3 or 4 at diagnosis, the use of more than 2 previous chemotherapy lines, over 20 months between diagnosis and transplant, CR pre-transplant status, LEAM vs. BEAM conditioning, the use of red cell transfusion, mucositis grade 3 or 4 post-transplant, because none of them reached statistical significance, we did not perform the multivariate analysis. Because there were few deaths at 100 day a univariate cox proportional hazards analysis would yield aberrant results, thus it was not performed. For the univariate analysis for RFS at 2 years we included age over 50, male sex, Hodgkin lymphoma diagnosis, Stage 3 or 4 at diagnosis, the use of more than 2 previous chemotherapy lines, over 20 months between diagnosis and transplant, CR pre-transplant status, BEAM vs. LEAM, or CLV conditioning, the use of red cell transfusion, mucositis grade 3 or 4 post-transplant, because none of them reached statistical significance, we did not perform the multivariate analysis. For the univariate analysis for OS at 2 years we included age over 50, male sex, Hodgkin lymphoma diagnosis, Stage 3 or 4 at diagnosis, the use of more than 2 previous chemotherapy lines, over 20 months between diagnosis and transplant, CR pre-transplant status, BEAM vs. LEAM, or CLV conditioning, the use of red cell transfusion, mucositis grade 3 or 4 post-transplant, from these, Hodgkin Lymphoma diagnosis and the use of red cell transfusion were statistically significant and remained statistically significant in the multivariate Cox proportional hazards model.

## Results

### Patient's Characteristics

The number of patients analyzed was 222 with age between 9 and 65 years old (median 36 years). There were 132 patients in BEAM group, 48 patients in LEAM group and 42 patients in CLV group.

### Hematological Toxicity

All patients developed grade IV neutropenia, anemia with/without transfusion necessity, severe thrombocytopenia with transfusion requirements in most cases. Only 2 patients (0.9%) that received BEAM conditioning regimen didn't require platelet transfusions. Table 3 presents the data regarding the hematological toxicity. Median time to platelet engraftment and neutrophil engraftment was 13 days (range) and 10 days (range), respectively.

**Table 3:** Hematological toxicities following conditioning chemotherapy.

**Conditioning**		**BEAM**	**LEAM**	**CLV**	***p*-value**
		***n*** **=** **132**	***n*** **=** **48**	***n*** **=** **42**	
Mean hemoglobin at start (± standard deviation) (g/dL)		12.19 (± 1.88)	11.95 (± 1.98)	12.60 (± 2.07)	0.363
Median minimum hemoglobin (quartile 1, quartile 3) (g/dL)		7.70 (7.00, 8.98)	7.60 (6.60, 8.50)	7.55 (6.88, 8.33)	0.395
Red cell transfusion	Yes	50	23	19	0.380
	No	82	25	23	
Median days of severe anemia (quartile 1, quartile 3)		1 (0, 6)	2 (0, 6)	3 (0, 5)	0.787
Median days of severe thrombocytopenia (quartile 1, quartile 3)		4 (3, 7)	4 (3, 8)	3.5 (2, 5)	0.186
Platelets transfusion	Yes	129	48	41	0.694
	No	2	0	0	
Median days of severe neutropenia (quartile 1, quartile 3)		7 (6, 8)	7 (6, 8)	7 (7, 8)	**0.0409**

*The bold values represent statistically significant values*.

**Figure 1:**
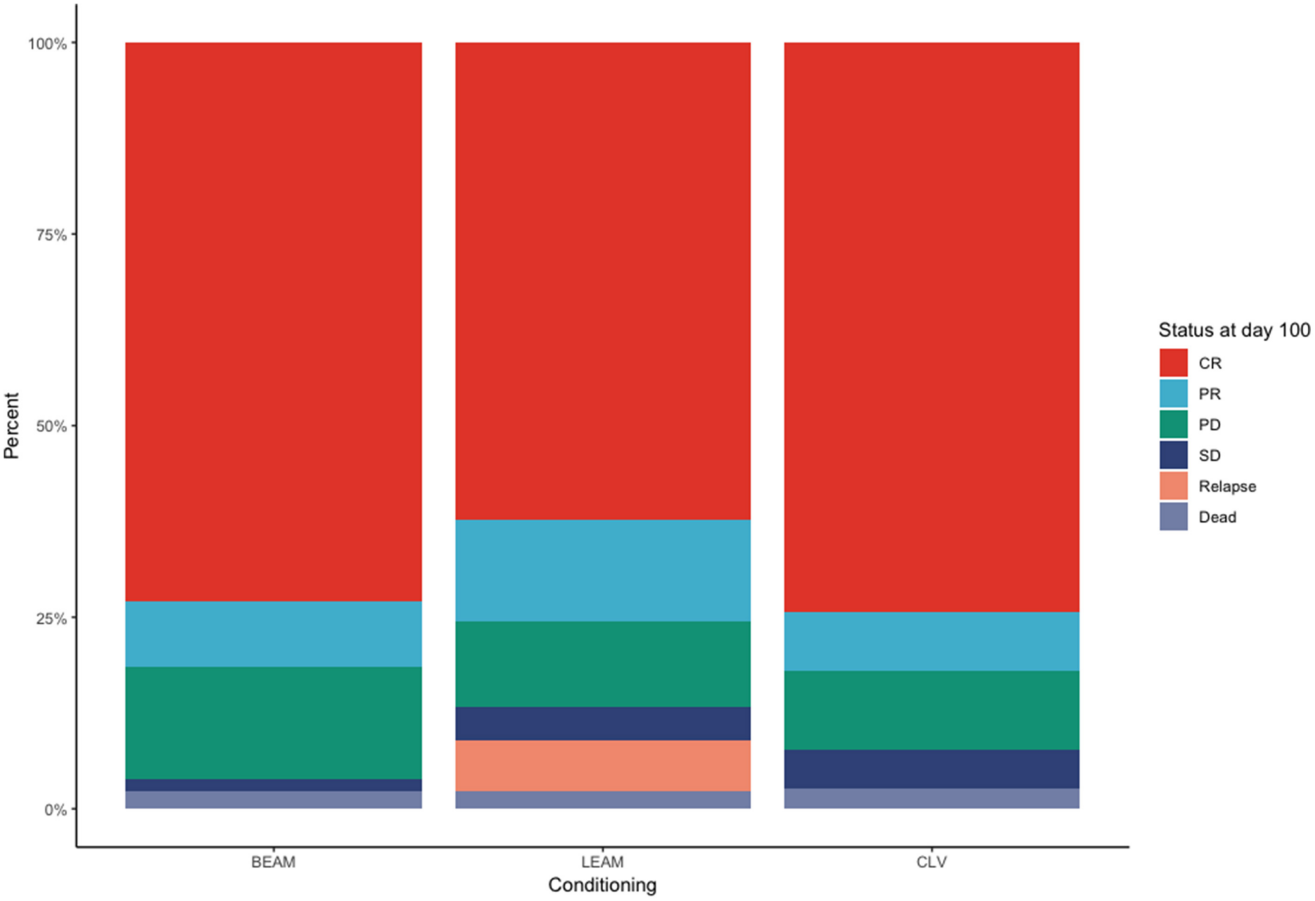
Status of the patients treated with the three conditioning chemotherapy regimens at day +100.

For the BEAM arm, two of the patients did not achieve platelet engraftment and died after transplant, whereas one patient didn't achieve neutrophil engraftment day 100 after transplant. Two patients developed as a complication hemophagocytic lymphohistiocytosis that led to delayed engraftment for platelets and neutrophils, until day 51, respectively, day 97 (Figure 1). For the LEAM arm, one patient died after transplant, before engrafting, and one patient didn't achieve platelet engraftment in day 100. For the CLV arm, one patient died after transplant, before engrafting.

### Extra-Hematological Toxicities

Gastrointestinal toxicity was the most common non-hematologic toxicity observed after all three conditioning regimens. Oral mucositis in various grades from I to IV was diagnosed in most cases. Other side effects include vomiting, diarrhea, colitis, and skin rash but with low severity grades (grade 1–2). Table 4 shows organ toxicity with clinical significance. From the BEAM arm, one patient developed grade 3 cardiac toxicity with sinus bradycardia and afterwards grade 4 with acute pulmonary edema, three patients presented a grade 3 pruritic skin rash and two patients developed grade 3 seizures. From the LEAM arm, one patient developed grade 3 upper gastrointestinal bleeding, one patient died (grade 5 toxicity) with acute renal failure, one patient developed hypoxic events up to grade 4 acute respiratory failure and one patient developed grade 3 itchy skin rash. From the CLV arm, one patient developed grade 3 colitis, one patient with grade 3 hepatic cytolysis, one patient with cardiac toxicity followed by death (grade 5) caused by an acute myocardial infarction with ST elevation and one patient with pulmonary toxicity clinically manifested with grade 3 pleurisy.

**Table 4:** Organ side-effects following conditioning chemotherapy.

**Conditioning**		**BEAM**	**LEAM**	**CLV**
		**(*n* = 132)**	**(*n* = 48)**	**(*n* = 42)**
Oral mucositis	3	40	15	11
	4	15	11	3
Digestive toxicity	3/4	6	1	1
	5	0	0	0
Hepatic toxicity	3/4	0	1	1
	5	0	0	0
Renal toxicity	3/4	0	1	0
	5	0	1	0
Cardiac toxicity	3/4	1	0	0
	5	0	0	1
Pulmonary toxicity	3.4	0	1	1
	5	0	0	0
Skin toxicity	3/4	3	1	0
	5	0	0	0
Other toxicity	3/4	2	0	0
	5	0	0	0

### Survival

**Figure 2:**
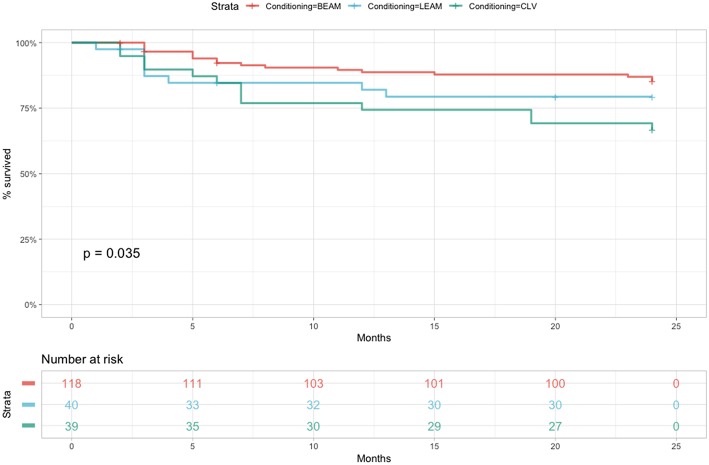
RFS comparing the three conditioning chemotherapy regimens at 2 years.

No statistical significance was observed in the univariate Cox proportional hazards model for relapse free survival until day 100. When considering RFS at 2 years (Figure 2), the only statistically significant difference was observed when comparing BEAM with LEAM or CLV (HR = 0.5, 95% CI 0.26–0.94, *p* = 0.031).

**Figure 3:**
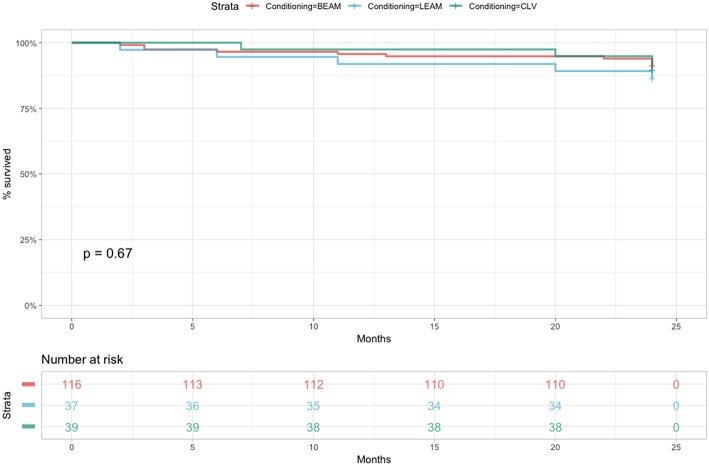
OS comparing the three conditioning chemotherapy regimens at 2 years.

When considering OS at 2 years (Figure 3), the statistically significant difference in survival was observed when comparing red blood cell transfusion (HR = 3.7, 95% CI 1.3–11, *p* = 0.013) and the diagnosis of Hodgkin Lymphoma (HR = 0.28, 95% CI 0.1–0.78, *p* = 0.015). Both the diagnosis of Hodgkin Lymphoma (HR = 0.34, 95% CI 0.12–0.96, *p* = 0.041) and red cell transfusion (HR = 3.58, 95% CI 1.26–10.17, *p* = 0.017) remained statistically significant in the multivariate Cox proportional hazards model. The Cox proportional hazard analysis is presented in Tables 5A–C.

**Table 5A:** RFS univariate analysis.

**Variable**	**HR**	**Lower 95% CI**	**Upper 95% CI**	***p*-value**
Age over 50	0.84	0.35	2	0.685
Male sex	0.81	0.43	1.5	0.516
HL diagnosis	1.1	0.57	2	0.83
Stage 3 or 4	1.6	0.72	3.7	0.237
More than 2 previous lines	0.82	0.43	1.6	0.548
Months until transplant over 20	0.65	0.34	1.3	0.202
Pretransplant status CR	0.67	0.35	1.3	0.217
BEAM vs. LEAM plus CLV	0.5	0.26	0.94	**0.031**
Red cell transfusion	1.1	0.55	2	0.874
Mucositis grade 3 or 4	1.4	0.75	2.7	0.291

*The bold values represent statistically significant values*.

**Table 5B:** OS univariate analysis.

**Variable**	**HR**	**Lower 95% CI**	**Upper 95% CI**	***p*-value**
Age over 50	2.4	0.9	6.2	0.082
Male sex	1.6	0.62	4.3	0.321
HL diagnosis	0.28	0.1	0.78	**0.015**
Stage 3 or 4	3.1	0.72	13	0.13
More than 2 previous lines	0.83	0.33	2.1	0.692
Months until transplant over 20	1.1	0.45	2.7	0.813
Pretransplant status CR	0.55	0.22	1.4	0.211
BEAM vs. LEAM plus CLV	0.72	0.29	1.8	0.475
Red cell transfusion	3.7	1.3	11	**0.013**
Mucositis grade 3 or 4	1.6	0.63	3.8	0.334

*The bold values represent statistically significant values*.

**Table 5c:** OS multivariate analysis.

**Variable**	**HR**	**Lower 95% CI**	**Upper 95% CI**	**p value**
HL diagnosis	0.34	0.12	0.96	**0.041**
Red cell transfusion	3.58	1.26	10.17	**0.017**

*The bold values represent statistically significant values*.

The TRM, hospitalization time and duration of antibiotics use are depicted in Table 6. For the BEAM arm, three patients died before day +100, all of them with sepsis, for the LEAM arm one patient died because of acute renal failure, whereas for the CLV arm, one died of acute myocardial infarction.

**Table 6:** TRM, hospitalization time and duration of antibiotics, for the three conditioning chemotherapy regimens.

**Conditioning**	**BEAM**	**LEAM**	**CLV**	***p*-value**
	**(*n* = 132)**	**(*n* = 48)**	**(*n* = 42)**	
Median antibiotic days (quartile 1, quartile 3)	7.00 (5.75, 10.00)	9.00 (7.00, 11.25)	8.50 (6.00, 11.75)	**0.0463**
Median hospitalization days (quartile 1, quartile 3)	22 (20, 24)	22 (21, 24)	22 (21, 25)	0.209
TRM (percent)	2.27	2.08	2.38	

*The bold values represent statistically significant values*.

## Discussions

In Europe over 8,000 ASCT for lymphomas were performed out of a total of 40,000 transplants according to the EBMT activity survey in 2017 (16). Retrospective and prospective studies have shown that PTCL patients who present response to initial chemotherapy and proceed to ASCT have a 4 years OS of 76–84% (5, 6). According to the MD Anderson Cancer Center (MDACC) experience, when using BEAM conditioning chemotherapy, both the OS and the PFS were higher in CR1. For CR2 and CR3, the OS were 59 and 53%. In Hodgkin's lymphoma, when comparing BEAM-based HDT to conventional chemotherapy as frontline treatment, patients with an advanced unfavorable HL that achieved CR or partial response (PR) after four courses of doxorubicin-containing regimens have a favorable outcome, with no benefit being shown from an early intensification with HDT and ASCT (17). Still, for relapsed/refractory (R/R) HL, ST followed by HDT and an ASCT is the standard of care, with a (18) F-fluordeoxyglucose (FDG) PET/CT intermediate negativity being the strongest predictor of clinical outcome (18). Choosing the best ST is difficult, being based on individual patient characteristics (19). ICE (ifosfamide, carboplatin, etoposide) is associated with 50% CR (20) and similar results are reported with DHAP and ESHAP (etoposide, methylprednisolone, high dose cytarabine and cisplatin), as well as with IGEV (ifosfamide, prednisolone, gemcitabine, and vinorelbine) (54% CR and 27% PR) (21–23).

Standard conditioning therapies for ASCT in lymphoma are currently thus BCNU-based, BEAM being the most commonly used conditioning regimen in Europe, while CBV is frequently used in North America. Shortage of BCNU is a world-wide problem determined by limited availability of the alcoholic solvent needed for its preparation, leading to the development of other conditioning regimens for ASCT in patients with lymphoma. Lomustine (CCNU) seemed the logical replacement of BCNU and resulted in the use of alternative regimens, as is the case for LEAM and CLV. Melphalan-related problems on market required the emergence of new conditioning regimens without melphalan (e.g., CLV, LACE (lomustine, cytarabine (Ara-C), cyclophosphamide, etoposide) (24–26). In our centers, we examined the toxicities of LEAM and CLV regimens, compared with standard BEAM.

Early data about high incidence of pulmonary toxicity (20–30%) associated to BCNU has driven the search for alternative regimens. Chao et al. have recommended a dose-escalation study with CCNU replacing BCNU in CBV regimen in 12 lymphoma patients and reported the maximum tolerated dose (MTD) of 15 mg/kg of CCNU orally (27, 28). At Stanford, a phase II study evaluating CLV vs. CBV conditioning in lymphoma patients reported the same efficacy but with higher pulmonary toxicity in the CLV regimen using 15 mg/kg CCNU (28), equivalent to more than 500 mg/m^2^. In a dose escalation prospective study performed on 14 patients, do Santos et al. have reported an MTD of 300 mg/m^2^ CCNU in combination with etoposide, cytarabine and melphalan as an alternative LEAM regimen (8). Most studies comparing the CCNU-based regimen with the standard BEAM used the dosage of 200 mg/m^2^ reporting similar efficacy and toxicity (5, 29, 30). Still, there are no randomized studies comparing standard BEAM conditioning with other preparative regimens for ASCT in lymphoma patients.

Our study compares engraftment, early toxicity and TRM, between the three conditioning regimens BEAM, LEAM and CLV for a cohort of 222 patients receiving ASCT over a period of 5 years. We did not find statistically significant differences between the three groups in parameters measuring immediate post-transplant outcome, as is the case for data regarding engraftment, therapy-related toxicity, antibiotic use, duration of hospitalization, 100 days TRM. The median time to neutrophil engraftment in our study was 10 days, comparable with data reported by Sharma et al. (14) and Kothari et al. (5). The median time for platelet engraftment in our study, of 13 days, was comparable with 12 days reported by Kothari et al. but shorter compared to the data, of 18.5 days, presented by Sharma et al. (Table 7). Still, when comparing our results with similar studies, major drawbacks are the differences in timepoints and demographic characteristics.

**Table 7:** Comparison of the toxicity results between previously published data and the Romanian cohort.

	**Sharma et al**. **(****14****)**		**Kulkarni et al**. **(****31****)**		**Kothari et al**. **(****5****)**		**Romanian society for bone marrow transplant study**		
**Total number of patients**	51		206		100		222		
	**BEAM**	**LEAM**	**BEAM**	**BEAM**	**BEAM**	**BEAM**	**BEAM**	**BEAM**	**CLV**
Number of patients/group	34 (66.6%)	17 (33.3%)	150 (72.8%)	56 (27.8%)	50 (50%)	50 (50%)	132 (59.45%)	48 (21.62%)	42 (18.9%)
PMN engraftment (median day)	12	15			11	11	10	10	10
PLT engraftment (median day)	18.5	22			12	12	13	13	13
Antibiotic days median	22	18					7	9	8.5
Median hospitalization days	20.5	25			23	23	22	22	22
OM grd III/IV	23 (68%)	11 (65%)					55 (41.6%)	16 (33.3%)	14 (33.33%)
**Digestive toxicity**									
3/4	16 (47%)	7 (41%)					6 (4.5%)	1 (2.08%)	1 (2.38%)
5							0	0	0
**Hepatic toxicity**									
3/4	NA	NA					0	1 (2.08%)	1 (2.38%)
5							0	0	0
**Renal toxicity**									
3/4	NA	NA					0	1 (2.08%	0
5							0	**1 (2.08%)**	0
**Cardiac toxicity**									
3/4	NA	NA					1 (0.75%)	0	0
5							0	0	**1 (2.38%)**
**Pulmonary toxicity**									
3/4	3 (9%)	1 (6%)					0	1 (2.08%)	1 (2.38%)
5							0	0	0
**Skin toxicity**									
3/4	NA	NA					3 (2.27%)	1 (2.08%)	0
5							0	0	0
**Other toxicity**									
3/4	NA	NA					2 (15%)	0	0
5							0	0	0
TRM (day 0–100)	6 (18%)	2 (12%)	4.67%	1.8%	2%	4%	2.27%	2.08%	2.38%
Deaths at 1 year			17.5%	14.3%					

*The bold values represent statistically significant values*.

The median hospitalization period for patients in the 3 groups in our study was 22 days, similar to data reported by other studies (5, 14), but we observed a shorter duration of antibiotic treatment compared with the Sharma et al. study. The most frequent grade 3/4 non-hematologic toxicity was oral mucositis with a higher incidence of 41.6% in the BEAM group, compared to 33.3% in both the LEAM and CLV groups, but with lower incidence than in other studies in both CCNU and BCNU based conditioning reporting (reporting 2/3 of cases) (14, 32).

A major concern of BCNU treatment was pulmonary toxicity reported in earlier studies (33, 34). Regimens containing high doses of CCNU also led to an important incidence of interstitial pneumonitis (IP) in up to 63% of cases (28). The use of lower doses of CCNU was associated with lower incidence of IP. Sharma et al. (14) compared LEAM vs. BEAM and reported 6% and, respectively, 9% grade III and IV pulmonary toxicity in the 2 groups. In our study, there were no cases with grade III/IV pulmonary toxicity in the BEAM group and 1 case in each group receiving LEAM and CLV (2.08 and 2.38%, respectively).

In our study TRM was similar in the three groups, with 3 infection related deaths in the BEAM group (TRM 2.27%) and 1 toxicity related death in each of the other two groups (LEAM- TRM 2.08% and CLV- TRM 2.38%). Our data are comparable with those reported by Kulkarni et al. (31) and Kothary et al. (5) and are significantly lower than those presented by the Sharma et al. (14).

The choice of HDT regimen before an ASCT for both HL and NHL is guided by limited data. In a large retrospective analysis of almost 5,000 patients treated over a 13-year period between 1995 and 2003, the study coordinated by Pasquini et al. on behalf of the Center for International Blood and Marrow Research (CIBMTR) have compared the conditioning chemotherapy regimens BEAM (1730 patients) to cyclophosphamide plus BCNU plus etoposide (CBV) (1853 patients), busulfan plus cyclophosphamide (789 patients) and to TBI-based treatment (545 patients) (35–40). The 1-year incidence of IPS was 3 to 6% and was highest in recipients of CBV and TBI, compared with BEAM. One-year TRM was 4 to 8%, respectively, and was similar between regimens. For NHL cases, there was a significant interaction between histology, HDT regimen, and outcome. Compared with BEAM, CBV was associated with lower mortality in follicular lymphoma and CBV was associated with higher mortality in diffuse large B cell lymphoma. For patients with HL, CBV, busulfan plus cyclophosphamide and TBI were associated with higher mortality compared with BEAM. They concluded thus that the impact of specific AHCT regimen on post-transplantation survival is different depending on histology. Therefore, further studies are required to define the best regimen for specific diseases, our study bringing new information for transplant physicians for R/R lymphomas that undergo an autologous stem cell transplantation.

## Conclusion

An autologous stem cell transplantation is the best therapeutic approach for a R/R lymphoma. Conditioning chemotherapy is of utmost importance, with LEAM, BEAM and CLV conditioning regimens being considered as viable alternatives. In this study we present the comparative analysis of a large cohort of patients with R/R lymphoma undergoing ASCT with BEAM/LEAM/CLV conditioning and we document the similar outcome regarding engraftment, early toxicity, and TRM. The data presented only analyses the clinical status of the patients in the first 100 days following the transplant and future studies should be carried out to compare the three conditioning regimens beyond day +100 after the transplant.

## Data Availability

All datasets generated for this study are included in the manuscript/supplementary files.

## Ethics Statement

The study was reviews and approved by the ethics committees of both the Fundeni Clinical Institute in Bucharest, as well as of the Coltea Hospital in Bucharest.

## Author Contributions

All authors have read and approved the manuscript and contributed to data gathering. SP did the statistics. HB, AncC, AndC, and CT wrote the manuscript. AT supervised the work.

### Conflict of Interest Statement

The authors declare that the research was conducted in the absence of any commercial or financial relationships that could be construed as a potential conflict of interest.
